# *Edwardsiella andrillae*, a New Species of Sea Anemone from Antarctic Ice

**DOI:** 10.1371/journal.pone.0083476

**Published:** 2013-12-11

**Authors:** Marymegan Daly, Frank Rack, Robert Zook

**Affiliations:** 1 Department of Evolution, Ecology & Organismal Biology, The Ohio State University, Columbus, Ohio, United States of America; 2 Antarctic Geological Drilling Science Management Office, University of Nebraska-Lincoln, Lincoln, Nebraska, United States of America; The Evergreen State College, United States of America

## Abstract

Exploration of the lower surface of the Ross Ice Shelf in Antarctica by the Submersible Capable of under-Ice Navigation and Imaging (SCINI) remotely operated vehicle discovered a new species of sea anemone living in this previously undocumented ecosystem. This discovery was a significant outcome of the Coulman High Project’s geophysical and environmental fieldwork in 2010-2011 as part of the ANDRILL (ANtarctic geologic DRILLing) program. *Edwardsiella andrillae* n. sp., lives with most of its column in the ice shelf, with only the tentacle crown extending into the seawater below. In addition to being the only Antarctic representative of the genus, *Edwardsiella andrillae* is distinguished from all other species of the genus in the number of tentacles and in the size and distribution of cnidae. The anatomy and histology of *Edwardsiella andrillae* present no features that explain how this animal withstands the challenges of life in such an unusual habitat.

## Introduction

**Figure 1:**
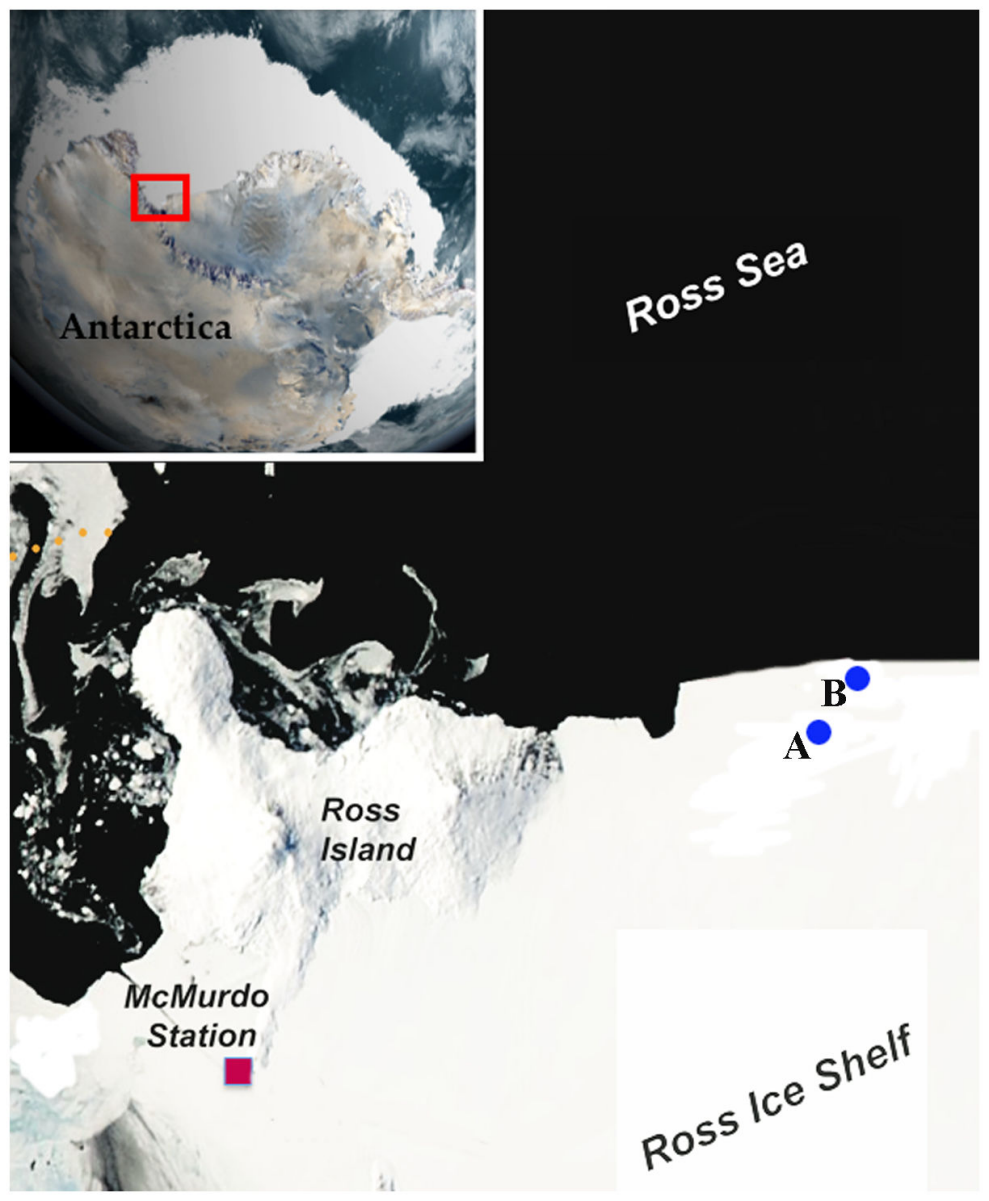
Known localities of *Edwardsiella andrillae*, n. sp. The site labeled A is at 77° 31.6’ S 171° 20.1’ E ; this corresponds to “Site 3” for the 2010-2011 SCINI dive series. The site labeled B is at 77° 28.03’ S 171° 36.28’ E ; this corresponds to “Site 4 (CH-1)” for the for the 2010-2011 SCINI dive series (Rack et al., 2012).

The biota associated with glacial ice is poorly documented because the habitat is largely inaccessible and is technologically difficult to access. As part of the multi-national ANtarctic geological DRILLing (ANDRILL) program, a remotely operated vehicle called the Submersible Capable of under-Ice Navigation and Imaging (SCINI) [1] was deployed from the Ross Ice Shelf (Figure 1) through a 30-cm hole drilled by a hot water drill at two distinct locations [2]. This provided an unexpected and astonishing glimpse into this subsurface world, discovering an unusual and likely unique marine biological community dominated by anemones living inside burrows in the lower surface of the ice shelf.

**Figure 2:**
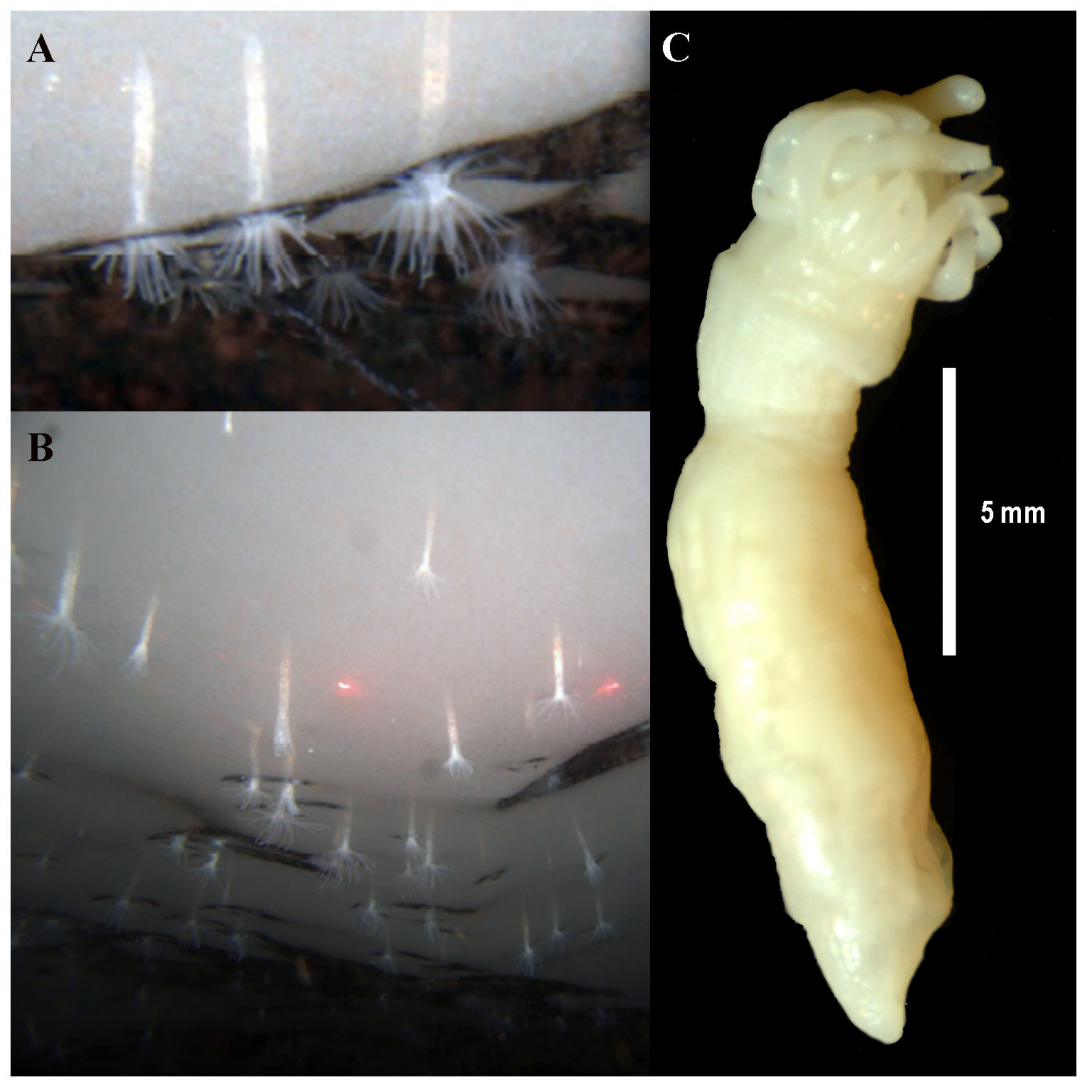
External anatomy and habitus of *Edwardsiella andrillae* n. sp. A. Close up of specimens *in* *situ*. Image captured by SCINI. B. “Field” of *Edwardsiella andrillae* n. sp. in situ. Image captured by SCINI. Red dots are 10 cm apart.

At 77° 31.6’ S 171° 20.1’ E (Figure 1, site A), the upward-facing cameras on SCINI captured images of a field of approximately 100 m^2^ inhabited by small, tentaculate animals living with most of their body inside the ice shelf, with tentacles dangling into the water below. A second field of animals was discovered approximately 6 km away at 77° 28.03’ S 171° 36.28’ E (Figure 1, site B). In both places, the animals appear similar in size and are spaced more or less uniformly (Figure 2A, B). The ice shelf is approximately 250-260 m thick at these sites; mean sea level below the ice shelf surface is approximately 40 m. 

These animals are sea anemones of a new species, here described as *Edwardsiella andrillae*. *Edwardsiella* is a genus of Edwardsiidae, a family of burrowing anemones reported from habitats ranging from the deepest trenches [3] to hypersaline [4] and hyposaline [5,6] coastal estuaries. All previously described species belonging to *Edwardsiella* are from coastal waters. 

This is the first species of sea anemone reported to live in ice. Previously described species of sea anemones from Antarctica are reported from hard [7-9] or soft [10-12] substrates, but always below the anchor ice. 

The unprecedented habitat of *Edwardsiella andrillae* raises questions about the biology, physiology, and life history of the animal that cannot be answered given the present material. The means by which these animals burrow into the ice shelf is unclear, as are the physiological mechanisms that enable them to live in ice. Burrowing by sea anemones has been described as a process of serial expansion and deflation of the pedal disc [11,13] or digging with the tentacles [10]; neither of these strategies would seem possible in solid ice. These animals are significantly larger than those reported from brine channels [14]. Annelid worms living in glaciers have physiological mechanisms including novel strategies for producing and using energy [16] and for stabilizing tubulin [17] to facilitate life at extremely low temperatures. As is the case in the ice worm *Mesenchytraeus solifugus* [17], the morphology of *Edwardsiella andrillae* does not suggest any adaptation to the unusual environment it inhabits. 

## Materials and Methods

Specimens were removed from the ice using an improvised suction sampler mounted on the outside of the SCINI remotely operated vehicle. The sampler consists of a plastic tube with an opening positioned within the SCINI forward camera’s field of view that is connected through a one-way valve to a water filter and chamber where the samples are collected and stored until the vehicle is recovered to the surface. An external, inverted tunnel thruster powered by the vehicle is connected to the distal end of the plastic tube and sampling chamber to provide water suction. The SCINI vehicle was flown under the ice shelf and positioned so that the tube opening was close to the seawater-ice interface and thus able to capture the organisms as they floated by or were extracted from their ice shelf burrows. Hot water from the drill system was pumped down from the surface of the ice shelf and used to flood the basal ice to stun the organisms and assist with the extraction process. Once the vehicle was recovered, the suction sampler was disassembled and the specimens were placed in ethanol for the helicopter trip back to McMurdo Station, where some samples were transferred to formalin for long-term preservation and further study. More than 20 samples were collected using this device mounted on the SCINI vehicle during a series of dives through the ice shelf.

### The samples were collected through the U.S. Antarctic Program (USAP) by Event G

75 049-M (PI = F. Rack) based on a permit request that was processed by the U.S. National Science Foundation (NSF) pursuant to the Antarctic Conservation Act as amended by the Antarctic Science, Tourism and Conservation Act (NSF Form 1078). NSF determined that no specific permit was required to collect marine anemones from under the Ross Ice Shelf at this location.

Whole formalin-fixed specimens were examined and photographed under a dissecting microscope. Four formalin-fixed specimens were dehydrated and embedded in paraffin, serially-sectioned at 10 µm, and stained in Heidenhain’s Azan [18]. Nematocyst preparations were made by cutting a small (>0.5 mm^2^) piece of tissue from each of two formalin-fixed specimens, floating this tissue in water on a microscope slide and then smashing and smearing the tissue with a coverslip. Because of the small size of specimens, sampling for cnidae was destructive and thus the number of samples examined is limited. Nematocyst measurements were made following [19] and capsules were identified following [20,21].

### Nomenclatural acts

The electronic edition of this article conforms to the requirements of the amended International Code of Zoological Nomenclature, and hence the new names contained herein are available under that Code from the electronic edition of this article. This published work and the nomenclatural acts it contains have been registered in ZooBank, the online registration system for the ICZN. The ZooBank LSIDs (Life Science Identifiers) can be resolved and the associated information viewed through any standard web browser by appending the LSID to the prefix "http://zoobank.org/". The LSID for this publication is: urn:lsid:zoobank.org:pub:BB12B7B1-89F4-4DE3-ADCA-66CE0EA8D149. The electronic edition of this work was published in a journal with an ISSN, and has been archived and is available from the following digital repositories: PubMed Central, LOCKSS. The holotype and paratypes have been deposited in the American Museum of Natural History. 

### Taxonomic treatment

Order Actiniaria

Family Edwardsiidae Andres, 1881

#### Definition.

Actiniaria with elongate, vermiform body usually divisible into two or more regions: between long scapus provided with periderm and short capitulum may be short scapulus lacking periderm and ectodermal specializations. Aboral end rounded, may be differentiated into physa. No sphincter or Acontia. Mesenteries divisible into macro- and micro-cnemes; always eight perfect macrocnemes and at least four microcnemes. Macrocnemes comprise two pairs of directives and four lateral mesenteries, two on each side, whose retractors face ventral directives. Retractors diffuse to strongly restricted; parietal muscles always distinct [19].

Genus *Edwardsiella* Andres, 1883

#### Definition.

Edwardsiidae with column clearly differentiated into capitulum and scapus. Three or more cycles of tentacles. Tentacles hexamerously arranged, those of innermost cycle longest. Capitulum ridged; nematocysts concentrated on ridges. Scapus generally bears periderm, always lacks nemathybomes or tenaculi. Aboral end rounded but not differentiated into a physa. Ciliated tracts of filaments short, discontinuous [22].

#### Type species.

*Edwardsia carnea* Gosse, 1856 by subsequent designation [23].

*Edwardsiella andrillae* n. sp.

**Figure 3:**
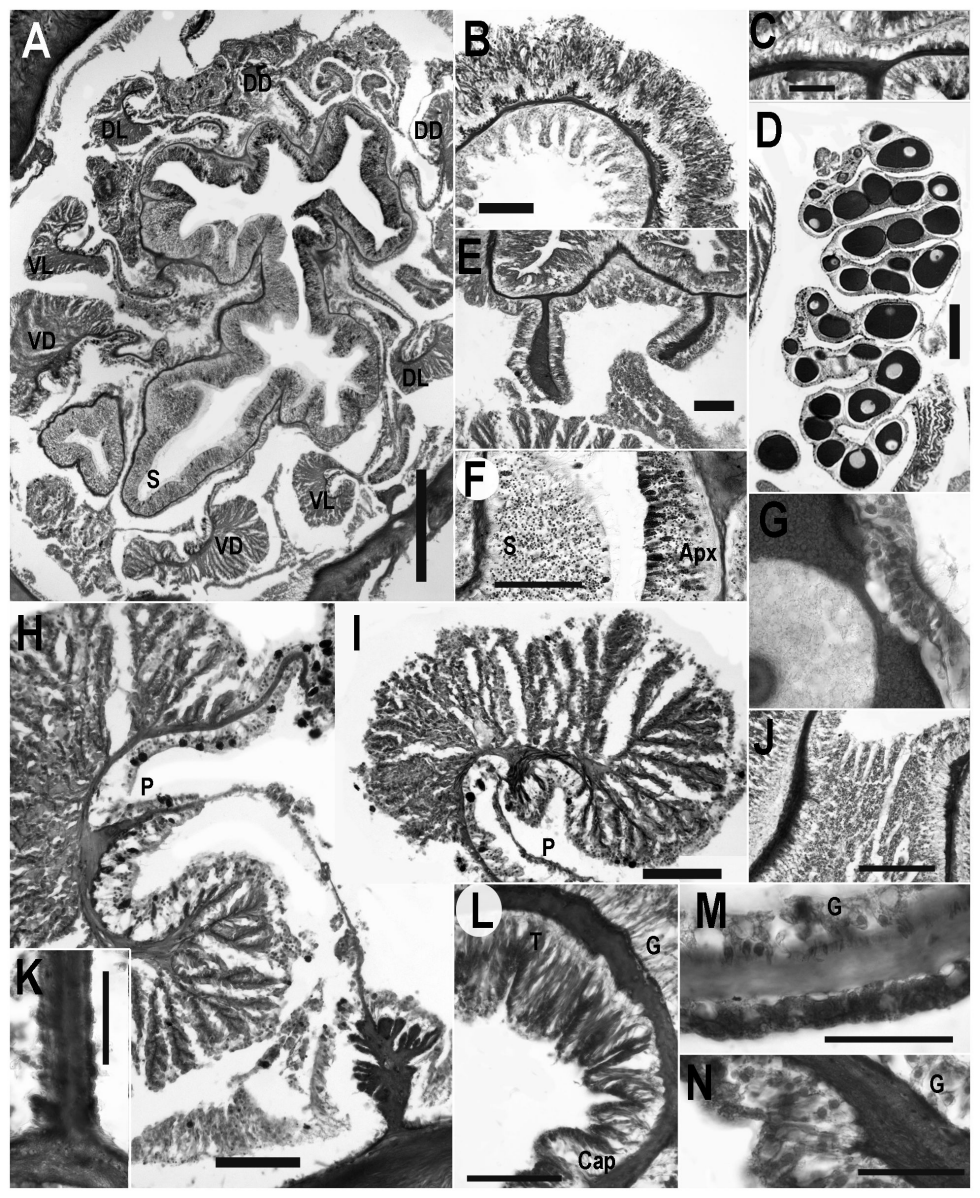
Internal anatomy and histology of *Edwardsiella andrillae* n. sp. All scale bars =100µm unless otherwise noted. A. Cross section through actinopharynx showing mesenteries and siphonoglyph. Scale = 500µm. B. Cross section through tentacle showing relatively strong ectodermal musculature and abundant spirocysts. C. Longitudinal section through oral disc showing relatively weak ectodermal musculature. Scale bar =20 µm. D. Gametogenic region of mesentery of female specimen. E. Cross section through distal column showing microcnemes. F. Close-up view of actinopharynx, showing histological differentiation of siphonoglyph. G. Trophonema of mature oocyte. Scale bar =30 µm. H. Retractor and parietal muscle of macrocnemic mesentery. I. Retractor muscle of Macrocneme. J. Musculature of base of tentacle. K. Junction between aboral end and mesentery. Note absence of basilar muscles. Scale bar =25 µm. L. Longitudinal section through distal column showing transition between tentacle and capitulum. M. Longitudinal section through scapus. Scale bar =30 µm. N. Longitudinal section through aboral end. Abbreviations: Apx, actinopharynx; Cap; capituluar ectoderm; DD, dorsal directive mesentery; DL, dorsolateral mesentery; G, gastrodermal side of body wall; P; junction of mesentery and retractor muscle; S, siphonoglyph; T, tentacle; VD, ventrolateral directive mesentery; VL, ventrolateral mesentery.

**Figure 4:**
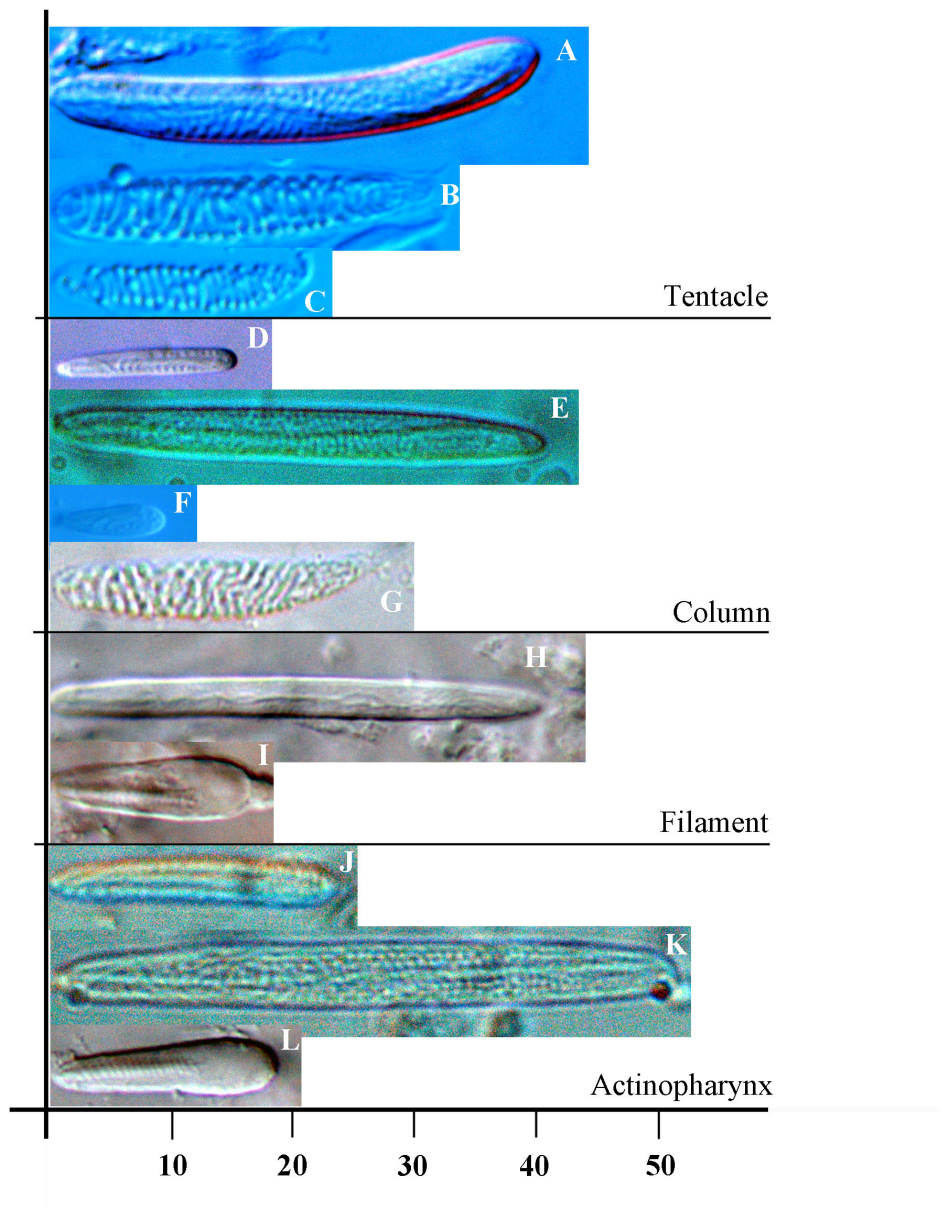
Cnidae of *Edwardsiella andrillae* n. sp. Scale at bottom, in µm, applies to all images. See Table 1 for size ranges for each capsule type in each tissue. A. Basitrich. B. Spirocyst. C. Spirocyst. Although this capsule is smaller and has a thinner tubule than the spirocyst in Figure 4A, spirocysts show continuous variation in capsule size and robustness. D. Small basitrich. E. Basitrich. F. Small microbasic mastigophore. The small size of these cnidae precludes distinguishing them as *b*- or *p*- mastigophores. G. Spirocyst. H. Basitrich. I. Microbasic *p*-mastigophore. J. Small basitrich. K. Basitrich. L. Microbasic *p*-mastigophores.

Figures 2-4; Table 1 urn:lsid:zoobank.org:act:42A55C7F-86B8-41F0-A6B6-2CD0221A8FF3

**Table 1:** Size range (in μm) of the cnidae of *Edwardsiella andrillae*.

	*Edwardsiella andrillae*	**S**	**N**	*Edwardsiella ignota* (from [30])
**Tentacles**				
Small basitrichs	none seen			17-19.7 x 3-3.5
Basitrichs (A)	(29.5) 33.1-46.6 (48.5) x 3.5-5.3	2:2	58	17-24 x 4.2-5.6
Spirocysts (B, C)	19.5-37.1 (39.9) x 2.9-6.3	2:2	62	none reported
**Column**				
Small basitrichs (D)	15.4-20.2 x 2.2-3.0	2:2	16	14-21.5 x 3.5-4.2
Basitrichs (E)	41.2-46.6 x 4.3-5.1	2:2	6	(22.6) 26.8- 36.7 x 5.6-8.5
Small mi. mastigophores (F)	9.9-13.7 x 2.4-4.0	2:2	22	none reported
Spirocysts (G)	24.0-32.4 x 3.9-6.0	2:2	9	none reported
**Filaments**				
Small basitrichs	none seen			15.5-19.7 x 2.5
Basitrichs (H)	16.0-24.7 x 2.3-3.5	3:3	33	22.6-25.4 x 3.5-5
Mi. *p*-mastigophores (I)	18.3-27.5 x (4.3) 4.3-6.7	3:3	65	18.3-24.0 x 3.5-5.6
**Actinopharynx**				
Small basitrichs (J)	20.0-31.3 x 2.0-3.1	2:2	14	15.5-19.7 x 2.8
Basitrichs (K)	39.6-54.3 x 3.0-5.3	2:2	35	none reported
Mi. *p*-mastigophores (L)	17.3-28.6 x 4.4-6.3	2:2	28	21.0-24.0 x 3.0-3.5

Specimens (S) indicates how many of the sampled specimens contained a particular type of cnida; Number (N) is the total number of capsules measured. The letter in parentheses after each type refers to Figure 4. Where multiple sizes of e.g., basitrichs are reported, the capsules within any specimen show discontinuous rather than overlapping size ranges. The small microbasic mastigophores of the column could not be distinguished as *p*- or *b-*mastigophores; these overlap in size with the small basitrichs but differ in capsule shape and tubule morphology (see Figure 4). Spirocysts are more common in the column than the measurement imply: many broken or partially discharged spirocysts were seen in the column samples. Abbreviation: Mi.= microbasic.

#### Diagnosis

*Edwardsiella* with tapering, elongate column and 20-24 tapering tentacles (Figure 2B); inner tentacles notably longer. Column and tentacles opaque white, without periderm. Length of column of whole contracted specimens 16-20 mm, column diameter to 6 mm. 

#### External anatomy

Column naked: no periderm or cuticle. In preserved specimens, capitulum short, same yellowish-white color as scapus, ridges faint; scapus long, smooth, tapers from widest point at junction with capitulum to slightly pointed aboral end (Figure 2). Regionation of the column not very pronounced. Capitulum not visible in most specimens because capitulum and base of tentacles contracted and pulled inside scapus. Mesenterial insertions visible as unbroken, straight furrows along length of column; highly contracted animals have deeper furrows than relaxed animals. Aboral end tapered rather than swollen or rounded, not differentiated from scapus; highly contracted individuals may have small pore at tip, suggesting that proximal-most part of aboral end is contracted inside the column. Tentacles in two crowded cycles differentiated by size: 8 tentacles in inner cycle longer, thicker than 12-16 tentacles of outer cycle. 

#### Internal anatomy and histology

Longitudinal muscles of tentacles (Figure 3B) and radial muscles of oral disc (Figure 3C) ectodermal; muscles of disc weaker than those of tentacles. Tentacle with endodermal musculature at junction with oral disc (Figure 3J). Column without marginal sphincter muscle (Figure 3L). Ectoderm of capitulum thicker, more columnar than that of scapus (compare Figure 3L, 3M); ectoderm of aboral end slightly thicker but otherwise not differentiated from scapus (Figure 3N).

Eight macrocnemes of equal size and development (Figure 3A) span length of column. Two pairs directives; ventral pair attaches to single, deep, ventral siphonoglyph (Figure 3A). Siphonoglyph glandular, without nematocysts; ectoderm of actinopharynx columnar, containing glandular cells and nematocysts (Figure 3F). At least four pairs of microscopic microcnemes (Figure 3E); microcnemes without muscles, reproductive tissue, filaments, extend less than 1 mm below tentacle, not visible in every specimen or section because of unequal contraction of specimens. 

Parietal muscle strongly restricted, with thick mesoglea and few, globular folds; muscle approximately equally developed on both surfaces but not symmetrical (Figure 3H). Macrocnemic mesenteries very thin between retractor and parietal muscle. Retractor muscles circumscribed, reniform, with many thin, highly branched folds (Figure 3I). Branches of retractor similar in height, widely spaced, with few ramifications. All macrocnemes fertile below region of actinopharynx; sexes apparently separate, only female specimens sectioned (Figure 3D). Eggs large (200-500 µm), yolky, with trophonema (Figures 3D, 3G). Basilar muscles absent; mesentery has microscopic expansion of mesoglea and slight fold at junction with aboral end (Figure 3K). 

Cnidom Spirocysts, basitrichs, microbasic *p*-mastigophores (Figure 4, Table 1).

#### Material examined

Specimens were observed at 77° 31.6’ S 171° 20.1’ E and 77° 28.03’ S 171° 36.28’ E (Figure 1A, B respectively). Samples collected from these two sites, within 50 m of the drill hole at the lower surface of the Ross Ice Shelf. Holotype: AMNH 5350, whole specimen, from 77° 31.6’ S 171° 20.1’ E (Figure 1, site A). Paratypes: AMNH 5351, two whole specimens, 77° 31.6’ S 171° 20.1’ E (Figure 1, site A); AMNH 5352, 16 cross-section histological slides, 77° 31.6’ S 171° 20.1’ E (Figure 1, site A); AMNH 5353, whole specimen, 77° 28.03’ S 171° 36.28’ E (Figure 1, site B); AMNH 5354, 13 longitudinal-section histological slides, 77° 28.03’ S 171° 36.28’ E (Figure 1, site B). 

#### Etymology

This species is named after the **An**tarctic **Dril**ling program that resulted in the collection of the specimens. 

## Discussion

Although most edwardsiids are burrowers in soft sediments [5,19,22,24], members of *Edwardsiella* also live in vegetation mats, in crevices, and in skeletons of dead *Lophelia* corals [23]. Unlike many other groups of anemones whose dispersal potential is limited, some members of *Edwardsiella* may disperse larger distances because of associations with other animals: members of *Edwardsiella carnea* (Gosse 1856) and *Edwardsiella lineata* (Verrill, 1873) parasitize ctenophores as juveniles, using the host for dispersal and food [5,25,26]. Such associations are not known for all species in the genus. 

Most species of *Edwardsiella* are described from the northern hemisphere [27]; only *Edwardsiella ignota* Carlgren 1959 has been reported from the southern hemisphere (Chile). *Edwardsiella andrillae* differs most notably from *Edwardsiella ignota* in cnidom (Table 1). The actiniarian fauna of Antarctica includes at least two other species of Edwardsiidae: *Edwardsia meridionalis* Williams 1981 and *Scolanthus intermedius* (McMurrich 1893). These differ from *Edwardsiella andrillae* in having nemathybomes, small batteries of nematocysts in the column ectoderm. Furthermore, *E. meridionalis* also has fewer tentacles [10], and *S. intermedius* has much smaller retractor muscles despite having a generally larger body size [25,26].

All eggs in the sectioned individuals are at approximately the same developmental stage (Figure 3D): some of these appear smaller (or larger) in section because the sections are tangential. All have a clearly defined nucleolus and are thus at the “late vitellogenic oocycte” stage as defined in [28]. This suggests that *Edwardsiella andrillae* undergoes seasonal rather than continuous reproduction, as is common in many Antarctic invertebrates [28]. 

The means by which *Edwardsiella andrillae* achieves it relatively large numbers is not clear. *Edwardsiella lineata* and the edwardsiid *Nematostella vectensis* Stephenson 1935 are able to reproduce asexually via transverse fission [29]; this can lead to large numbers of coincident individuals. Even edwardsiids that are not known to undergo asexual reproduction can achieve high densities, through high recruitment, low dispersal, or unrecognized asexual reproduction: *Edwardsia meridionalis* occurs at densities in excess of 10,000 individuals per square meter in waters 20-65 m deep in Antarctica (Cape Bird, New Harbor, and the jetty off McMurdo Station: [8]) and *Edwardsia isimangaliso* Daly et al. 2012 and *Edwardsia elegans* Verrill 1869 can achieve densities in the tens to hundreds of individuals per meter [4], (MD pers obs). Although not testable with the present material, these alternatives can be distinguished because they predict different population genetics and demography: Low larval dispersal or high larval recruitment would lead to genetically heterogeneous populations of individuals at many sizes or developmental stages, whereas asexual reproduction would lead to genetically homogenous populations of individuals of the same size or developmental stage. 
